# THE RELATIONSHIP OF SOCIAL ISOLATION TO SELF-NEGLECT AMONG OLDER ADULTS: RESULTS OF A NATIONAL SURVEY

**DOI:** 10.1093/geroni/igad104.1559

**Published:** 2023-12-21

**Authors:** Ashwin Kotwal, Rebecca Mullen, Irena Cenzer, Jason Burnett, Carla Perissinotto, Louise Hawkley

**Affiliations:** University of California San Francisco, San Francisco, California, United States; University of Colorado, Aurora, Colorado, United States; University of California San Francisco, San Francisco, California, United States; University of Texas Health Science Center at Houston, Houston, Texas, United States; University of California San Francisco, San Francisco, California, United States; NORC at the University of Chicago, Chicago, Illinois, United States

## Abstract

Self-neglect among older adults is characterized by inattention to hygiene and one’s immediate living conditions, and may reflect unmet needs from social relationships. We therefore determined if social isolation was associated with self-neglect and how the association differed by gender. We used data from the National Social Life, Health, and Aging Project (NSHAP) Wave 3 (2015), a nationally-representative survey of 3,677 community-dwelling older adults. Social isolation was determined using a 12-item scale assessing household contacts, social network interaction, and community engagement. Self-neglect was assessed in-person and included 1) body neglect (lowest quintile of bodily self-presentation related to clothes and hygiene) and 2) household neglect (lowest quintile of household building condition, cleanliness, odor and clutter). Logistic regression was used to determine the adjusted probability of self-neglect by social isolation, and interaction terms with gender. Results indicated the association between social isolation and self-neglect differed by gender (p-values for interaction: body neglect: 0.02, household neglect: 0.20). Among women, social isolation was associated with a higher risk of body neglect (social isolation: 26% vs no isolation: 14%, p=0.001) and household neglect (23% vs 17%, p=0.05). For men, social isolation was not associated with body neglect (27% vs 23% p=0.2) or household neglect (23% vs 22%, p=0.8). In summary, social isolation was associated with body and household neglect among women, but was not associated with neglect among men. Future work should investigate mechanisms for gender differences and interventions to address or prevent self-neglect through enhancing social connectedness.

